# Subgroup Formation in Diverse Virtual Teams: The Moderating Role of Identity Leadership

**DOI:** 10.3389/fpsyg.2021.722650

**Published:** 2021-10-21

**Authors:** Helen op ‘t Roodt, Henning Krug, Kathleen Otto

**Affiliations:** Department of Work and Organizational Psychology, Institute of Psychology, Philipps University of Marburg, Marburg, Germany

**Keywords:** virtual teams, identity leadership, e-leadership, diversity, subgroup formation, performance, team satisfaction

## Abstract

**Background:** As today’s organizations are becoming increasingly globalized and adding the impetus to a more remote form of working due to the present COVID-19 pandemic, new ways of collaboration—like virtual teams—have gained importance. In the present study, we aim to investigate how virtual team outcomes are linked to perceived diversity and subgroup formation and attempt to gain some initial insight into the role of the social identity approach to leadership in virtual teams.

**Method:** In the present cross-sectional study, a total of 102 virtual team members participated in an online survey measuring perceived diversity, identity leadership, subgroup formation, perceived performance, and team satisfaction, to examine the factors moderating the relationship between perceived diversity and subgroup formation as well as between perceived diversity and team performance and satisfaction.

**Results:** Moderation analysis revealed that perceived diversity had a negative influence on performance ratings when subgroups were highly perceived to be present, but not if subgroup formation was rated as low. The relationship between perceived diversity and team satisfaction was not moderated by perceived subgroup formation. Furthermore, identity leadership was found to be positively related to team satisfaction and perceived performance, while subjective diversity was negatively associated with both team outcomes. Identity leadership moderated the relationship between perceived diversity and subgroup formation, in that high levels of identity leadership weakened the positive relationship.

**Conclusion:** This study provides first evidence to the importance of the team leader’s role as a manager of a shared social identity in virtual teams where perceived differences can lead to subgroup splits, as identity leaders may hinder the emergence of subgroups in virtual teams.

## Introduction

Driven by increasing global competition and due to fast technological advancements, organizations are attempting to become more adaptive in terms of new and more flexible work arrangements. The current COVID-19 pandemic has accelerated these developments and transformed many workplaces so that working remotely has become the “new normal” in many organizations (Hofmann et al., 2020). Even before the pandemic, interactions among people working together as a team to achieve organizational goals increasingly shifted from face-to-face interactions to interactions *via information and communication technologies* (ICT; Wärzner et al., 2017). Work teams that rely primarily on ICT are defined as *virtual teams*. More specifically, virtual teams are characterized as groups consisting of “geographically, organizationally and/or time dispersed workers brought together by information and telecommunication technologies to accomplish one or more organizational tasks” (Powell et al., 2004, p. 7).

Compared to face-to-face teams, working in a virtual team poses additional challenges due to the geographical dispersion of virtual team members and increased difficulties to build strong emotional ties and share knowledge among virtual teammates while communicating *via* ICT (see Furumo and Pearson, 2006; Rosen et al., 2007; Morrison-Smith and Ruiz, 2020). We like to propose that a leader’s central task therefore is to establish a feeling of “us” to enable satisfying and efficient virtual collaboration. Notably, as team members are often spread out across various countries, they tend to be heterogeneous in their composition. Based on social identity and self-categorization theorizing, the perception of differences among team members may lead to the formation of subgroups, which is most likely the case when team members feel similar to and identify more strongly with only one part of the group based on some relevant attributes, while feeling dissimilar to others (e.g., Polzer et al., 2006; Au and Marks, 2012). Considering the potential risks of subgroup formation in virtual teams related to social processes and performance-related outcomes (e.g., Polzer et al., 2006), further examination of factors impeding the formation of subgroups in the virtual environment is warranted and therefore addressed in the present study (see Gilson et al., 2015). As virtual teams have become more prominent in today’s organizations (Hofmann et al., 2020), this study aims to contribute to a better understanding of its potential pitfalls by focusing on the role of perceived diversity and the formation of subgroups within virtual teams and the associated relational and performance outcomes. Specifically, we aim to examine the role of a potentially alleviating factor in the relationship between perceived diversity and subgroup formation, namely the team leader and his or her ability to create a shared sense of identity (i.e., identity leadership; Steffens et al., 2014b; Haslam et al., 2020).

## Diversity, Subgroup Formation, and Leadership in Virtual Teams

In the following, we will take a closer look at two of the most frequently mentioned challenges of virtual teams, which go hand in hand with the geographic dispersion which virtual teams commonly face: Diversity and subgroup formation (e.g., O’Leary and Mortensen, 2010; Gilson et al., 2015; Morrison-Smith and Ruiz, 2020).

### Perceived Diversity and Diversity Effects in Virtual Teams

Diversity is conceptualized as general differences between people on the surface or deep-level that can cause individuals to perceive themselves as being different to another person (van Knippenberg and Schippers, 2007; Batarseh et al., 2017). Virtual teams, especially if global, are more heterogeneous compared to face-to-face teams in terms of both surface-level (i.e., readily observable characteristics like age and skin color) and deep-level aspects (i.e., non-observable traits like opinions and attitudes), as they frequently consist of members from different nations, speaking different native languages, and living in distinct time zones; hence, they are also more likely to differ in their cultural backgrounds and norms (Gibson et al., 2014; Han and Beyerlein, 2016).

In previous research about virtual team diversity, a wide range of diversity dimensions have been examined, like cultural, nationality, or functional diversity (e.g., Staples and Zhao, 2006; Shachaf, 2008; Peters and Karren, 2009; Batarseh et al., 2017). In the present study, we draw attention away from one or more specific dimensions of diversity and focus on a more general conceptualization of diversity, namely perceived diversity in terms of the degree to which an individual perceives and is aware of differences within a collective (Hentschel et al., 2013; see Shemla et al., 2016, for a review). The main idea behind examining this awareness-related form of perceived diversity is that actual differences are frequently unrelated to perceptions of differences among group members. The use of ICT with only limited opportunities to exchange interpersonal cues raises the question of whether and what dimension of (actual) diversity team members are aware of (Harrison et al., 2002; Hentschel et al., 2013). Even if diversity is objectively present in a work team, members might not be aware of these differences or each member might perceive them differently, for example, due to different cultural backgrounds that attach different importance to certain attributes (Shemla and Meyer, 2012). Hence, individuals’ perceptions are oftentimes a better predictor of diversity effects and performance (Wayne and Liden, 1995; Harrison et al., 2002; Allen et al., 2008; Hentschel et al., 2013).

Literature shows both positive and negative aspects of team diversity, highlighting the benefits of diverse knowledge and experiences when it comes to the elaboration of information for decision-making and innovation processes (see van Knippenberg et al., 2004; Shachaf, 2008; Batarseh et al., 2017; Carter and Phillips, 2017), but also the limiting effects of misunderstandings or ineffective communication (Gibson and Gibbs, 2006; Shachaf, 2008), which can interfere with performance (e.g., Han and Beyerlein, 2016). Recently, scholars have shown that problems in diverse virtually operating groups are, among others, ineffective coordination of tasks, less identification with and integration of the team members into the group as well as weaker relational ties and conflicts between the members (e.g., Hinds and Mortensen, 2005; Stahl et al., 2010). Au and Marks (2012) showed that perceived differences between virtual team members regarding their national backgrounds made it more difficult for the team members to build a common identity. Similarities as opposed to differences, on the other side, help establishing a shared understanding in virtually operating teams through means like shared experiences, direct communication, and information exchange (Hinds and Weisband, 2003). According to *similarity-attraction theory* (Byrne, 1971), team members’ perception of being similar to others in terms of one or more characteristics can lead to social attraction, leading to higher levels of trust and facilitating the formation of close relationships (i.e., Turban and Jones, 1988; Jimenez et al., 2017). Differences among the team members, however, can activate intergroup bias and cause in-group vs. out-group categorization, resulting in subgroup splits (Tajfel and Turner, 1979, 1986) while people favor their own in-group, leading to less trustful relationships within the virtual team and coordination difficulties (e.g., O’Leary and Mortensen, 2010; Robert, 2015).

### Subgroup Formation in Virtual Teams

Diversity has often been linked to the formation of subgroups in previous research, which frequently has been identified as a negative predictor of virtual team outcomes and processes (e.g., Polzer et al., 2006; O’Leary and Mortensen, 2010; Gibbs et al., 2017). Subgroups appear when smaller groups within teams are formed, mostly when hypothetical dividing lines based on one or more salient attributes exist (Lau and Murnighan, 1998; Thatcher and Patel, 2011). Subgroup processes are often described in terms of the *social identity approach*, comprising *social identity theory* (Tajfel and Turner, 1979) and *self-categorization theory* (Turner et al., 1987). This is because the formation of subgroups often originates in subgroup identification processes, with members of a subunit feeling more similar to each other than to the group as a whole (Salk and Brannen, 2000; Carton and Cummings, 2012).

*Social identity theory* (Tajfel, 1974; Tajfel and Turner, 1979) states that individuals derive their self-concept not only from their *personal identity* (“I”), which rests on individual characteristics, interests, features, and values, but also from their *social identity* (“we”), which is based on group memberships and group prototypes (Brewer and Gardner, 1996; Brickson, 2013). Context and salient cues determine whether personal identity guides behavior or rather one of the individuals’ (activated) social identities (Hewstone et al., 2002). *Self-categorization theory* (Turner et al., 1987; Turner, 2010; Turner and Reynolds, 2011) is an extension of social identity theory and states that people use external cues to categorize one another. While people different to oneself regarding characteristics like demographic variables (like gender, nationality, or ethnic background) tend to be classified as out-group members, more similar people to oneself are categorized as in-group members (e.g., Chatman and Spataro, 2005). Being a member of a particular group and strongly identifying with this group predicts intergroup bias, marked by positive attitudes and more collegial behaviors towards the own in-group (i.e., in-group favoritism) compared to negative or even hostile evaluations of and behaviors towards the out-group (Tajfel and Turner, 1979; see also Zenger and Lawrence, 1989; Tyler and Blader, 2001).

The development of small groups within a team can cause conflicts and less trust among group members, decreased cohesion and identification on the socio-emotional level (e.g., Polzer et al., 2006; Newell et al., 2007; O’Leary and Mortensen, 2010; Paul et al., 2016). When it comes to organizing tasks, difficulties in coordination and exchanging information across existing subgroups can hinder performance (e.g., Lau and Murnighan, 2005; Meyer and Schermuly, 2012). The perception of being different from some team members, while identifying with others, can trigger self-categorization processes in virtual teams that culminate in us-vs.-them thinking (e.g., Yilmaz and Peña, 2014). The social identity approach thus poses an explanation for an individual’s conduct and attitude towards their teammates and can further explain why subgroups might emerge in virtual teams. But what can managers and team leaders do to get the best out of the heterogeneous composition of their virtual team, so that subgroups are less likely to occur?

### Leaders as Identity Creators in Virtual Teams: The Social Identity Approach to Leadership

Team leadership is a particularly important driver of success in virtual teams (e.g., Garro-Abarca et al., 2021). The use of ICT implies the need to acquire the right e-leadership competencies like knowledge about the communication strategies and media, intercultural skills, and technological skills to facilitate collaboration (van Wart et al., 2019; Contreras et al., 2020). In the present study, we want to focus on the social identity approach to leadership that builds on the principles of social identity theory and self-categorization theory (Haslam et al., 2020). This approach highlights the importance of a leader’s ability to motivate others to work toward a group goal by representing the group, developing a shared understanding of what “we” stand for and thus promoting a sense of shared identity (Ellemers et al., 2004; Hogg et al., 2012; Steffens et al., 2014b; Haslam et al., 2020). Leadership, thus, is described as a social process in which leaders influence followers through establishing a feeling of unity and identification with the group they are leading. Influence processes as well as leader–follower interactions are thereby determined by the degree to which a common identity is established (Epitropaki et al., 2017). To establish a shared understanding of “us,” leaders engage in social identity management behaviors. This form of leadership has been conceptualized as *identity leadership* consisting of four dimensions, that are identity prototypicality, identity entrepreneurship, identity advancement, and identity impresarioship (Steffens et al., 2014b). *Identity prototypicality* is the degree to which the leader is perceived to incorporate the norms and values and thereby represents specific features of the group (i.e., the leader is seen as “being one of us”; Steffens et al., 2014b, p. 1003; Haslam et al., 2020). *Identity entrepreneurship* (i.e., “crafting a sense of us”; Steffens et al., 2014b, p. 1004) refers to a leader’s behaviors directed at establishing a cohesive tie among the group members, making them feel part of a superordinate “we” and influencing the members’ beliefs of what the groups incorporate and stand for (Reicher et al., 2005; Haslam et al., 2020). Moreover, identity leaders engage in *identity advancement* (i.e., “doing it for us”; Steffens et al., 2014b, p. 1003), which are behaviors targeted at advancing the interests of the group and defending the common interests in case they are at risk. In this regard, it should be obvious that the leader is acting *for* the group and the collective goal, and not pursuing personal objectives or goals that might favor another group (Haslam et al., 2020). Before group members are to act for a specific shared goal, it is crucial for them to have internalized the values and norms regarding actions and behaviors. On these grounds, leaders act to emphasize the importance of the team (i.e., by “making us matter,” Steffens et al., 2014b, p. 1004), by displaying behavior known as *identity impresarioship*.

There are a range of reasons why we expect leaders engaging in social identity management to be beneficial to virtual team success. Earlier research has highlighted the need to build trust and establish a sense of cohesion in order to build an effective virtual team (e.g., Chinowsky and Rojas, 2003; Clark et al., 2010; Gazor, 2012). Highly identifying with one’s virtual team was shown to help overcome the perception of subgroups if faultlines were present (e.g., Boyraz, 2019), while a shared identity was also argued to foster effective communication and information sharing which is important to effectively work on common tasks (Kimble, 2011). Siebdrat et al. (2009) suggest that creating identification within the virtual team can help overcome issues such as conflict, which in turn leads to higher performance. In this vein, one best practice frequently mentioned is the implementation of regular team building activities or opportunities to have informal conversations (e.g., Wiesenfeld et al., 1998; Boule, 2008; Ellis et al., 2008). Following this argumentation, virtual team leaders should take on the task of implementing tactics that promote identification in order to create a common ground beneficial for effective virtual collaboration (see Sivunen, 2006).

## The Present Study

**Figure 1:**
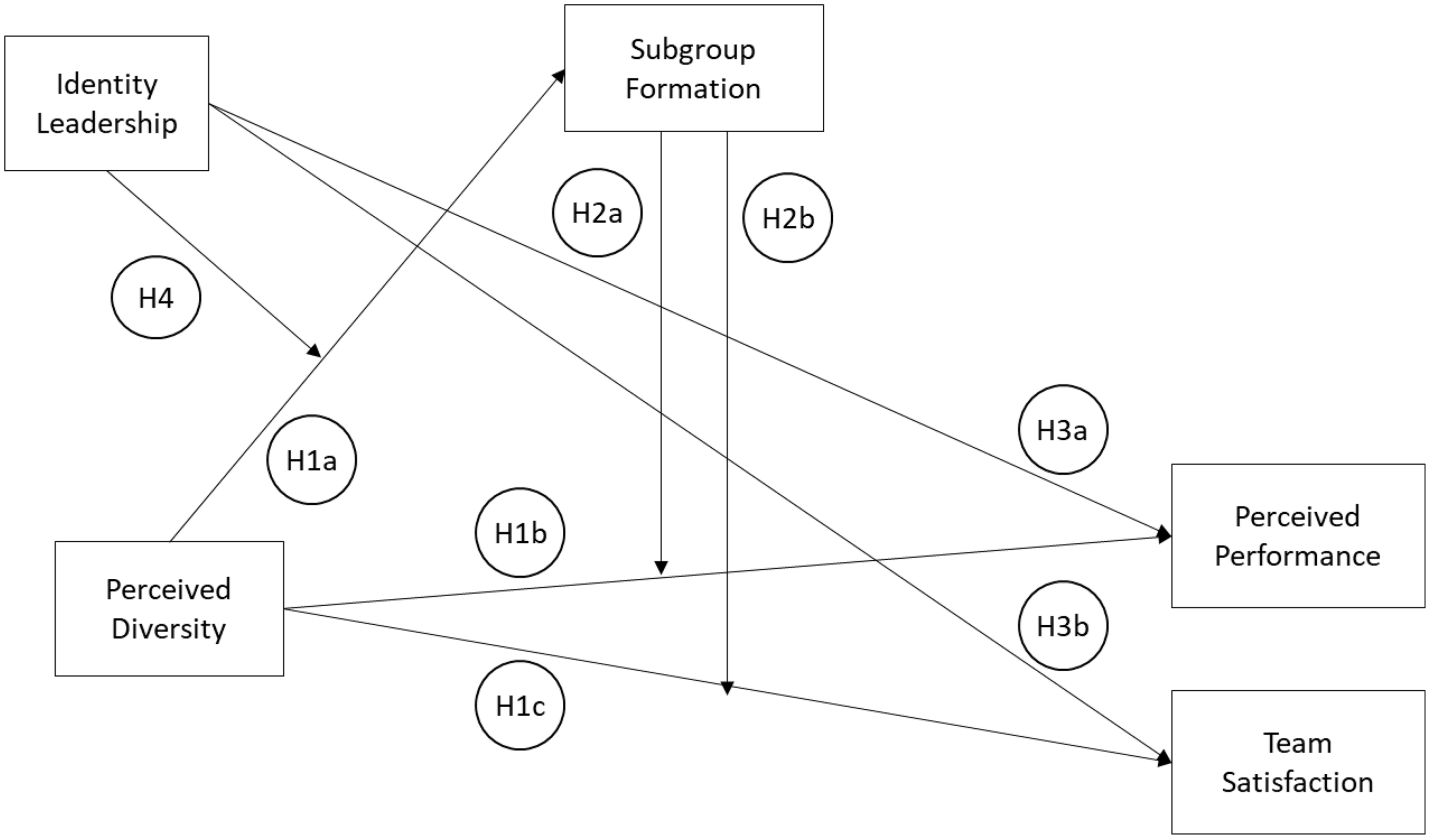
Proposed relationships among the selected variables.

The present cross-sectional study aims to examine the impact of perceived differences in virtual teams on the formation of subgroups and consequently on team satisfaction as well as perceived performance. Furthermore, the study focusses on the role of a potentially beneficial influence counteracting subgroup emergence in the light of high perceived diversity, namely the role of the team leader and their ability to establish a shared sense of identity also known as identity leadership. As the development of a shared sense of identity within the virtual team is argued to act as a “glue” (Fiol and O’Connor, 2005, p. 19) which binds team members together as a team so that subgroups are less likely to arise (e.g., Boyraz, 2019), the current study represents the first attempt to address the issue of identity leadership and the role it plays in the relationship between subjective team diversity and subgroup formation. Given the importance of the leader’s role and behavior to the success of virtual teams (Gibbs et al., 2017), further research on effective leader behavior, particularly in the context of the formation of subgroups in virtual teams, is warranted. Now that the main constructs examined in the study have been defined and contextualized with the virtual environment, a detailed derivation of the study’s hypotheses is presented. The proposed hypotheses and theoretical framework are presented in Figure 1.

### Perceived Diversity Hypotheses

In their special issue, Jimenez et al. (2017) summed up common challenges of global virtual teams and especially identified team diversity related challenges. In this context, subjectively perceived diversity was mentioned to hamper the development of interpersonal trust while communicating using ICT (Crisp and Jarvenpaa, 2013). According to *similarity-attraction theory* (Byrne, 1971), people prefer to interact and thus build relationships with similar as opposed to dissimilar others (Abrams and Hogg, 2006). A more diverse team constellation, on the other side, impedes social integration (Harrison et al., 2002), the development of an effective collaboration and communication climate and can lead to higher levels of conflict (Williams and O’Reilly, 1998; Kankanhalli et al., 2006; Wickramasinghe and Nandula, 2015). Mutual similarity can positively affect both number and quality of interactions required to coordinate efforts and finish tasks (see Bell, 2007; Guéguen et al., 2011). As social categorization theory (Turner et al., 1987) posits, the perception and awareness of differences can trigger categorization processes, as some of the members perceive themselves to be more similar to others, giving rise to ingroup–outgroup categorization. As a result, these salient differences serve as the basis for identifying with smaller groups (Hentschel et al., 2013), and subgroups might emerge. We thus propose that being aware of differences entails a negative evaluation of team outcomes, so that both satisfaction and perceived performance are lowered in those teams, in which members are highly aware of their heterogeneity, and that perceived diversity furthermore implies the perception of subgroup formation:

*H1a: Perceived diversity is positively associated with subgroup formation.*

*H1b: Perceived diversity is negatively associated with perceived team performance.*

*H1c: Perceived diversity is negatively associated with team satisfaction.*

As described earlier, diversity itself has been shown to lead to a wide range of team outcomes, both positive and negative. Subgroup emergence was mostly assumed to have detrimental effects on team functioning, when they are based on differences among team members regarding some attributes which trigger subgroup identification processes (Carton and Cummings, 2012; Robert, 2015). Earlier studies have shown that minimal categorical cues can lead to in-group favoritism and devaluing out-groups among virtual team members (e.g., Yilmaz and Peña, 2014). Similarities and differences are mostly subject to bad performance indicators if social categories are formed, and in-group members exchange more information with each other while out-group members are perceived to be less trustworthy and not all information is shared with them (Brewer, 1979; Polzer et al., 2006; Batarseh et al., 2017). Furthermore, Jehn and Bezrukova (2010) showed that perceived subgroup existence was positively associated with conflicts and coalition formation, leading to a decrease in team performance and satisfaction. We thus hypothesize:

*H2a: Perceived subgroup formation moderates the (negative) relationship between perceived diversity and perceived team performance, in that the relationship is weaker when team members rate subgroup formation in their team to be low.*

*H2b: Perceived subgroup formation moderates the (negative) relationship between perceived diversity and team satisfaction, in that the relationship is weaker when team members rate subgroup formation in their team to be low.*

### Identity Leadership Hypotheses

In virtual teams, many researchers have emphasized the importance of virtual leaders in contributing to positive team outcomes (e.g., Hoch and Dulebohn, 2017; Liao, 2017), as the team leader’s behavior also determines their team member’s behavior and commitment to the team goals (e.g., Darvish and Rezaei, 2011; Chai et al., 2017). As described earlier, the leader’s engagement in social identity management is intertwined with the capacities to shape and promote a shared collective identity in the group they are leading. When the team leader succeeds in making the shared purpose evident to all members, the leader awakens the followers’ desire to contribute their efforts to the attainment of the collective goal (e.g., Reicher et al., 2007; Steffens et al., 2014b). A common understanding of the group and its purpose is likely to lead to better team performance (e.g., Lurey and Raisinghani, 2001; Horwitz et al., 2006), while increased performance, in turn, is expected to translate into higher performance ratings. In addition, a heightened feeling of belonging within the team is likely to lead to a favorable team environment, which in turn might also influence the affective evaluation of the satisfaction of the team members with their co-workers (e.g., Serban and Roberts, 2016). Previous research has shown that the more team members perceived that their leader was acting in terms of social identity principles, the higher their engagement and perceived performance (e.g., Steffens et al., 2014a), commitment and motivation (e.g., Mertens et al., 2020) or employee health and well-being (Steffens et al., 2018) in a range of different contexts. Thus, consistent with the empirical and theoretical reasoning, we assume that the virtual team members’ perception of the team leader promoting the unity of the team has a positive impact on the team member’s attitudes toward their teammates and their performance evaluations. Our hypotheses can therefore be stated as follows:

*H3a: Identity leadership in virtual teams is positively associated with perceived team performance.*

*H3b: Identity leadership in virtual teams is positively associated with team satisfaction.*

The way diversity is dealt with in the team determines whether it has positive or negative effects on a wide range of team outcomes (see Gibbs et al., 2017). Increased identification is a powerful resource within a team and can enhance collaboration and cooperative behaviors by aligning the teams’ goals to the individuals’ goals so that members are more likely to contribute their efforts on behalf of the team (e.g., van der Vegt et al., 2003; Bezrukova et al., 2009). A strong team identity has been shown to be a moderating factor in the relationship between geographic dispersion and conflicts in distributed teams in that it lowers interpersonal conflict if team members are highly dispersed (Hinds and Mortensen, 2005). The role of fostering a shared sense of group belonging has been assigned to leaders who act according to social identity principles: Social identity leaders focus on the “we,” shifting the focus away from the individual perspective to the team as a whole (Steffens et al., 2014b; Haslam et al., 2020). The leader is meant to provide structures which help creating a feeling of belonging among the team members and build strong bonds among them, so that subgroups might not be perceived as strongly. We thus suggest:

*H4: Identity leadership moderates the relationship between perceived diversity and subgroup formation, in that the (positive) relationship is weaker when team members rate identity leadership to be high.*

## Materials and Methods

### Participants

For data collection, the study invitation link was posted in 10 social media groups and sent to 200 small- to medium-sized companies from various industries and countries, which described on their websites that they employ virtual or distributed teams. Participants were offered the chance to take part in a raffle and win one of nine Amazon gift cards. As a further incentive, participants interested in the research findings were sent a summary of the results. Responses were collected from September 23 until December 23, 2020.

Data from 102 individuals was collected, all passing the participation check questions (i.e., at least one member of the team is spatially separated from the rest of the team, the team meets less than once a month face-to-face, it consists of at least three members and members communicate at least 50% of the time using ICT). The sample consists of more men (*n*=58) than women (*n*=42), while two participants did not indicate their gender. Participants had an average age of 34.3years (*SD*=10.2). Most of the participants spoke Spanish as a native language (33.3%), followed by English (24.5%) and German (21.6%). Participants of 29 different nationalities are represented in the sample, while most of them were German (23.5%), followed by Argentinian (14.7%) and US-American (10.7%). The average team size was 8.3 members per virtual team (*SD*=4.9). Most of the participating team members were from the Computer, Software, and IT sector (59.8%). Participants were instructed to generate team codes to identify members from the same virtual team. Of four teams, at least 50% of the team members filled out the survey (which makes up a total of 15 participants).

The reliability of the employed measurements was evaluated using Cronbach’s alpha and greatest lower bound (glb). Note that due to missing values, the sample size varies from *N*=96 to *N*=102.

#### Perceived Team Diversity

In the present study, perceived diversity was measured by asking participants about general differences they observe to be salient within the group, without asking for special dimensions of diversity (see Shemla et al., 2016). The three-item measure developed by Hentschel et al. (2013) was used to examine perceived team diversity. The participants had to rate the extent to which they perceived differences within the virtual team to be salient (e.g., “When I am supposed to describe my work team, I automatically think about differences among my team colleagues”). The items were rated on a seven-point Likert-type scale, ranging from *strongly disagree* (1) to *strongly agree* (7). Internal consistency of the scale was indicated as good (*α*=0.80, glb=0.80, *N*=101).

#### Subgroup Formation

To measure team members’ perception of subgroup existence, a four-item scale developed by Cronin et al. (2011) was applied. An example item is “To what extent has your team split into subgroups?” Scales ranging from *not at all* (1) to *very much* (5) were used. The internal consistency of the scale was acceptable to good (*α*=0.71, glb=0.80, *N*=96).

#### Identity Leadership

The short form of the ILI (Steffens et al., 2014b) was used to assess the degree to which the leader is perceived to engage in social identity management. It consists of four items, reflecting each of the four dimensions of identity leadership. One sample item of the scale used is “The leader is a model member of the group” (identity prototypicality). Participants responded on a seven-point Likert-type scale, with response options ranging from *strongly disagree* (1) to *strongly agree* (7). The internal consistency of the ILI was very good, with Cronbach’s *α* of 0.89 and glb of 0.92 (*N*=99).

#### Team Outcomes (Team Performance and Team Satisfaction)

Perceived team performance was assessed by the question “How would you rate the overall quality of work done by your work group?” (Whitford et al., 2010) and rated on a five-point scale, ranging from *very poor* to *very good*, with *N*=101.

Consistent with other research, team satisfaction was measured by the three items used by van der Vegt et al. (2001; e.g., “I am satisfied with my present colleagues.”). Items were rated on a scale ranging from *strongly disagree* (1) to *strongly agree* (7). Reliability of the Team Satisfaction scale was high, *α*=0.92, glb=0.92, *N*=102.

#### Control Variables

As prior research has suggested, team size and task interdependence were assessed as control variables (see Robert, 2015; Boyraz, 2019). Team size was assessed with the question “If you consider the size of your primary team, how many team members do you have (excluding your team leader)?” (Algesheimer et al., 2011; the part in brackets was added in the present survey). Participants had to indicate the number of team members by typing in the total number. The degree of task interdependence among team members was measured by the five-item subscale of reciprocal independence developed by Pearce and Gregersen (1991). One sample item is “I frequently must coordinate my efforts with others.” Participants responded on a five-point scale ranging from *strongly disagree* (1) to *strongly agree* (5). Cronbach’s *α* was 0.85 and glb statistic was 0.88, indicating good internal consistency (*N*=102).

### Data Analysis Procedure

All analyses were conducted using R 3.6.3 (R Core Team, 2013). An analysis at the team level was not possible because of the lack of responses from a sufficient number of members of the same team. Therefore, individual level analyses were conducted. To test H1a, H1b, H1c, H3a, and H3b, bivariate correlations were calculated. H2a, H2b, and H4 were tested using moderated regression analyses while controlling for team size and task interdependence. To identify statistical significance, *p*<0.05 was used. For moderated regression analyses, predictor variables were mean centered by subtracting the mean of the variable (Aguinis and Gottfredson, 2010). For each of the regression models, it was screened for multivariate outliers using the distance measures Mahalanobis distance, Cook’s distance and leverage points (Tabachnick and Fidell, 2014; see Kannan and Manoj, 2015, for a comparison of distance measures). The cutoff score for leverage points was (2×*k*+2)/*N* and for Cook’s distance, the cutoff score of 4/(*N*−*k*−1) was employed, while *k* is the number of predictors and *N* the number of participants. For Mahalanobis distance, a value is considered a multivariate outlier if *p*<0.001. Data were excluded that had at least two multivariate outlier indicators.

## Results

Assumptions for calculating multiple regression models with interactions (i.e., linearity, normality, homoscedasticity) were checked and found satisfactory for all assumptions except from the normality assumption. Measure of multivariate skewness and kurtosis of Mardia (1970) reached significance (*p*<0.001), indicating that the multivariate distributions were significantly non-normal. However, moderation analysis was shown to be relatively robust to non-normality if the sample size is sufficiently big (*N*>30), which is the case in the present sample (Hayes, 2018). Variance Inflation Factors (VIF) were used to test for multicollinearity, and it was found that the VIF values were all below 2.19, and thus not indicative of serious multicollinearity problems (Alin, 2010). Missing values were present in the dataset (maximum 5.9% in one column or item). Multiple imputation was used for such rows and columns in the dataset in which less than 5% of data used for hypothesis testing was missing. Listwise deletion was employed for each subset of variables used for the analysis at hand, to not lose statistical power when excluding values which are not relevant for the respective analysis. Therefore, the resulting sample sizes vary across analyses and are noted.

### Correlational Analysis

Table 1 shows the intercorrelations of the study’s constructs as well as the variables’ means and standard deviations. For hypothesis testing, Pearson product-moment correlation coefficients are reported. Results are based on *N*=96. There was no positive relationship found between perceived diversity and subgroup formation (*r*=0.01, *p*=0.93), discarding H1a. Negative associations between perceived diversity and performance (*r*=−0.27, *p*=0.009) and perceived diversity and team satisfaction (*r*=−0.35, *p*<0.001) were found, thus supporting H1b and H1c. As proposed in H3a and H3b, identity leadership was shown to be significantly positively related to performance (*r*=0.42, *p*<0.001) and team satisfaction (*r*=0.54, *p*<0.001).

**Table 1:** Mean, standard deviations, and Pearson-moment correlations of the study’s constructs (*N*=96).

Variable	*M*	SD	1	2	3	4	5	6
Identity leadership	5.67	1.21	—					
Subgroup formation	2.89	0.79	−0.27^**^	—				
Performance	4.36	0.62	0.42^***^	−0.08	—			
Team satisfaction	5.99	1.00	0.54^***^	−0.23^*^	0.69^***^	—		
Perceived diversity	4.38	1.47	−0.07	0.01	−0.27^**^	−0.35^***^	—	
Size	8.70	6.41	−0.17	0.13	−0.17	−0.23^*^	0.07	—
Task interdependence	4.15	0.84	0.01	−0.12	0.08	0.07	−0.02	0.10

*p < 0.05; **p < 0.01; ***p < 0.001.

### Prediction of Team Performance and Team Satisfaction

**Figure 2:**
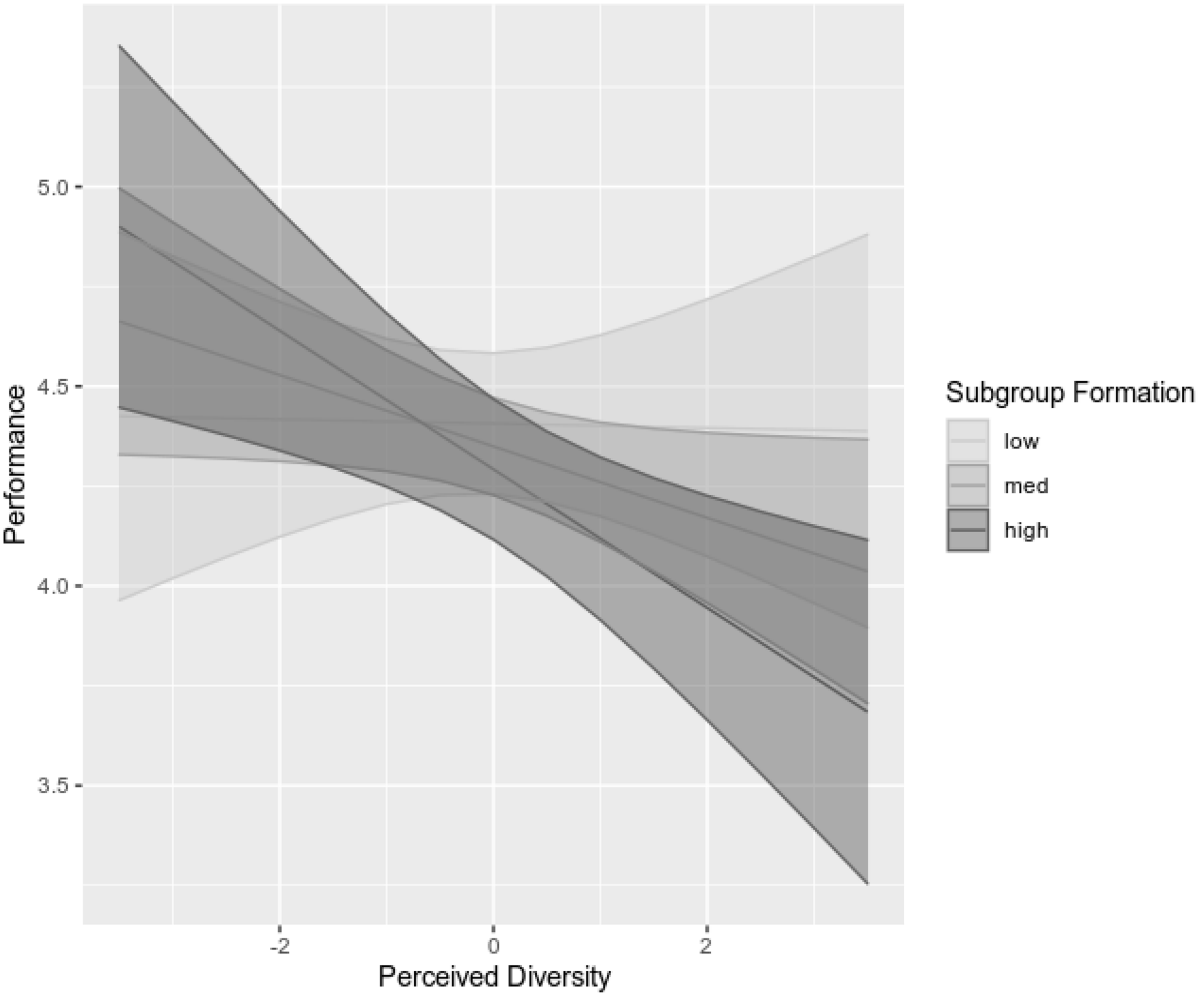
The relationship between perceived diversity and performance at low, medium, and high levels of subgroup formation. 95%-confidence intervals are depicted around the slopes.

Due to four missing values and three multivariate outliers, the data sample to predict perceived performance consisted of 95 participants. To test H2a, the control variables team size and task interdependence were entered into the model next to the main effects (perceived diversity and subgroup formation) and interaction term (see Table 2). The interaction term was negative and significant (*B*=−0.11, *p*=0.048). Simple slope analysis with one standard deviation above and below the mean of subgroup formation indicated that the relationship between perceived diversity and perceived performance was significantly negative when subgroups were highly perceived to have emerged (*B*=−0.17, *p*=0.004), but not when subgroups were less perceived (*B*=−0.00, *p*=0.951, see Figure 2). The entire regression model was significant, *R*^2^=0.15, *R*^2^_Adjusted_=0.10, *F*(5, 89)=3.14, *p*=0.012.

**Table 2:** Moderated regression results for perceived performance (*N*=95) and team satisfaction (*N*=96).

Predictor	Perceived performance					Team satisfaction				
	*B*	SE *B*	95% CI for *B*		*p*	*B*	SE *B*	95% CI for *B*		*p*
			Lower	Upper				Lower	Upper	
Intercept	4.09	0.35	3.39	4.78	<0.001	6.13	0.51	5.12	7.14	<0.001
Size	−0.02	0.01	−0.04	0.01	0.174	−0.02	0.02	−0.06	0.02	0.292
TI	0.10	0.08	−0.06	0.26	0.223	0.01	0.12	−0.22	0.24	0.938
PD	−0.09	0.04	−0.18	0.00	0.050	−0.26	0.07	−0.39	−0.13	<0.001
SF	−0.08	0.08	−0.24	0.09	0.373	−0.25	0.13	−0.50	0.01	0.057
PD×SF	−0.11	0.06	−0.22	−0.00	0.048	−0.13	0.08	−0.30	0.04	0.136

CI, confidence interval; TI, task interdependence; PD, perceived diversity scores; SF, subgroup formation scores. Perceived diversity and subgroup formation scores were mean centered.

The same variables were used to predict team satisfaction. There were three missing values and three multivariate outliers; hence, the analysis was conducted with *N*=96. As can be seen in Table 2, and against expectations (H2b), the relationship between perceived diversity and team satisfaction was not moderated by perceived subgroup existence (*B*=−0.13, *p*=0.136, *R*^2^=0.24, *R*^2^_Adjusted_=0.19, *F*(5, 90)=5.60, *p*<0.001). Only perceived diversity was directly and negatively related to team satisfaction, *B*=−0.26, *p*<0.001.

### Identity Leadership as a Moderator

**Figure 3:**
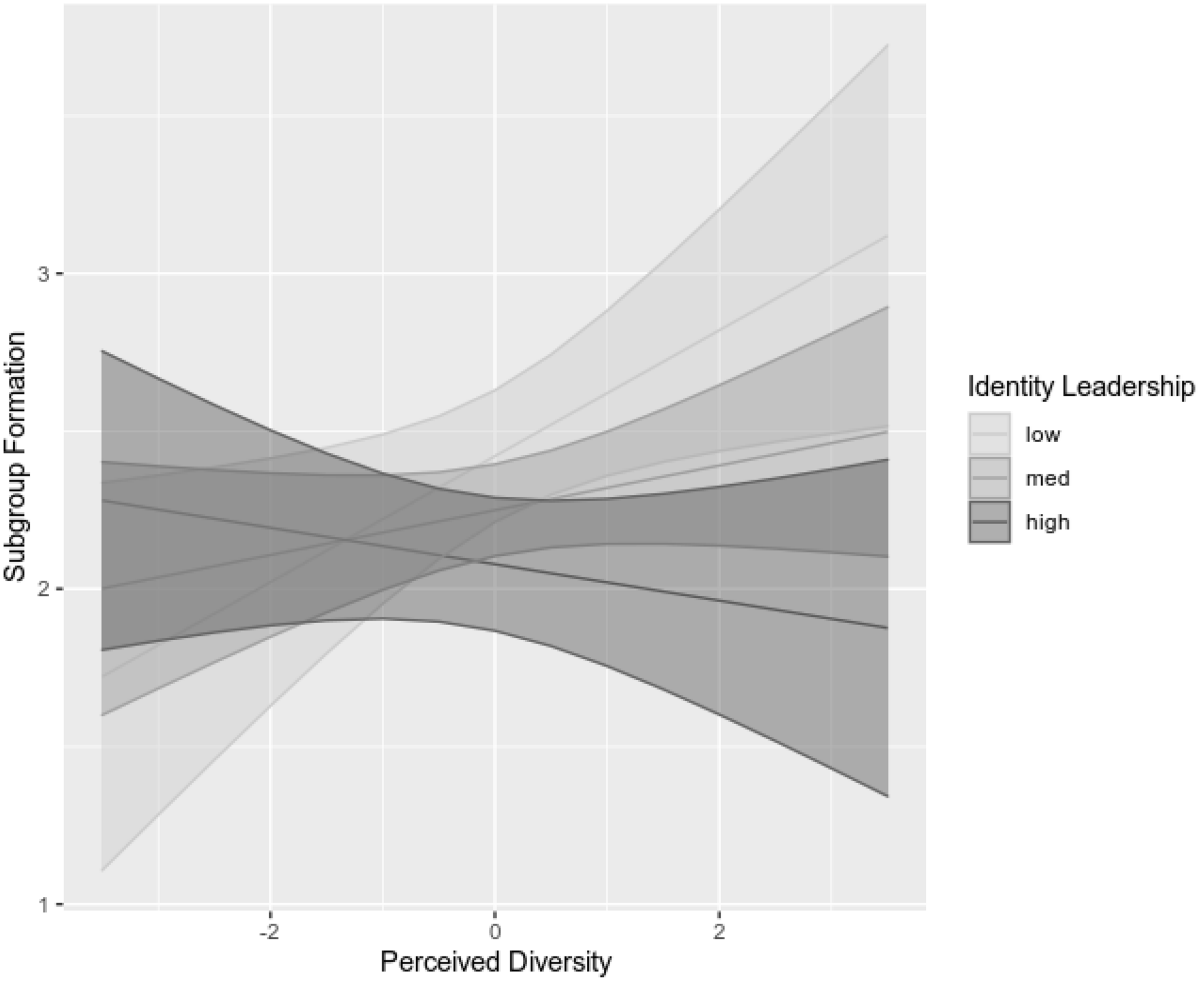
The relationship between perceived diversity and subgroup formation at low, medium, and high levels of identity leadership. 95%-confidence intervals are depicted around the slopes.

Due to five missing values, and after excluding data from two participants as they resulted outliers, analysis was performed with *N*=95. Regression coefficients can be found in Table 3. The relationship between perceived diversity and subgroup formation was moderated by identity leadership (*B*=−0.11, *p*=0.015). Overall, the regression model was significant, *F*(5, 89)=3.73, *p*=0.004, *R*^2^=0.17, *R*^2^_Adjusted_=0.13. Simple slope analysis was conducted to confirm the regression results (Aiken and West, 1991). The conditional values for identity leadership (one standard deviation above and below the mean; Cohen et al., 2003) were calculated. Simple slope analysis supported H4, showing that in teams where the leader was not evaluated to be an identity leader (low), a positive relationship between perceived diversity and subgroup formation emerged (*B*=0.20, *p*=0.017). In teams in which the leader was perceived to be an identity leader (high), perceived diversity did not significantly predict subgroup formation (*B*=−0.06, *p*=0.39). As depicted in Figure 3, at low levels of identity leadership, increased perceived diversity was associated with higher levels of subgroup formation. At high levels of identity leadership, increased perceived diversity was not related to subgroup formation.

**Table 3:** Regression coefficients of the moderated regression analysis predicting subgroup formation.

Predictor	*B*	SE *B*	95% CI for *B*		*p*
			Lower	Upper	
Intercept	2.06	0.41	1.25	2.86	<0.001
Size	0.03	0.02	0.00	0.06	0.040
Interdependence	−0.02	0.09	−0.20	0.17	0.849
Perceived diversity	0.07	0.05	−0.03	0.18	0.181
Identity leadership	−0.14	0.06	−0.27	−0.02	0.027
Identity leadership×Perceived diversity	−0.11	0.04	−0.19	−0.02	0.015

CI, confidence interval. Identity leadership and perceived diversity scores were mean centered.

## Discussion

The purpose of the present study was to investigate the impact of perceived diversity in virtual teams, on both team satisfaction and perceived performance. To determine under which circumstances perceived diversity has a negative influence on team outcomes, we examined subgroup formation as a moderating factor. In addition, this study aimed to gain further insights into the relationship between perceived diversity and subgroup formation by examining the moderating role of identity leadership.

The hypotheses are partly supported on basis of the present data. In line with our assumption, results indicate that perceived diversity is negatively related to both perceived performance and team satisfaction, thus supporting H1b and H1c. Contrary to expectations, perceived diversity was not significantly associated with subgroup formation (H1a). Thus, being highly aware of differences among the virtual team colleagues does not appear to be directly related to stronger perceptions of subgroups. Whether subgroups form or not when members are aware of their differences depends in part on whether team members conceive the entire team as a source of identification. Gaertner et al. (1996) have shown that the establishment of a shared identity and emphasizing a superordinate identity among subgroup members can help that team members categorize dissimilar individuals as part of the in-group, so that negative evaluations of dissimilar members are less likely (Tajfel and Turner, 1979).

Furthermore, as posed in H2a, participants indicating that subgroups are an issue in their team rate performance lower in highly perceived-to-be diverse teams, while heightened perceived diversity does not have a negative impact on performance in teams with low levels of subgroup formation. This result supports the widely accepted notion that diversity does not necessarily lead to negative performance outcomes. If intergroup biases are not activated, earlier research has found that diversity can unlock its potential through enhancing information elaboration, and the rich number of diverse resources and knowledge can positively contribute to problem solving and team performance (van Knippenberg et al., 2004; Carter and Phillips, 2017). On the other hand, if intergroup bias is activated, virtual team performance is impeded by higher levels of conflict, negative perceptions of the out-group, and sharing less information with out-group members (see Salk and Shenkar, 2001; Li and Hambrick, 2005; Pearsall et al., 2008; Jehn and Bezrukova, 2010; Paul et al., 2016).

Regarding team satisfaction, perceived subgroup formation is not shown to moderate the relationship between perceived diversity and team satisfaction, hence, discarding H2b. Satisfaction is rather directly and negatively influenced by perceived diversity, while subgroup formation did not significantly predict team satisfaction. The negative association between perceived diversity and team satisfaction can be explicated with similarity-attraction theory (Byrne, 1971), which offers an explanation why individuals prefer to interact and collaborate with teammates similar to themselves. Being highly aware of differences, however, is rather associated with feeling dissimilar as opposed to similar to their virtual team colleagues, irrespective of actual differences. The present finding regarding the role of perceived subgroup formation as a moderator in the relationship between diversity and team satisfaction diverges from the findings of Boyraz (2019), who showed that in teams with low perceived subgroups, faultlines were positively associated with team satisfaction. In her study, however, Boyraz did not investigate the influence of perceived diversity on team satisfaction, but how the relationship between team faultlines or the overlap between certain demographic variables (i.e., gender, location, function, and organizational tenure) and team satisfaction are influenced by perceived subgroup formation. While it is possible that dormant faultlines are not observed by team members, our measure of perceived diversity used in the present study directly implied that team members were aware of differences.

Our findings further indicate that virtual team members’ evaluation of the leader as an identity leader seems to play a valuable role in virtually cooperating teams: as predicted, identity leadership is positively associated with performance (H3a) and team satisfaction (H3b). As mentioned above, such leaders who are perceived to act in accordance with social identity principles can help overcome the formation of subgroups in teams where differences among members are strongly perceived, thus supporting H4. Only for participants who rated their leader as barely (as opposed to strongly) engaging in identity leadership were higher levels of perceived diversity associated with stronger perceptions of the existence of subgroups. This is consistent with social identity theory (Tajfel and Turner, 1979) and self-categorization theory (Turner et al., 1987), as leaders who develop and represent what the team stands for and foster a shared sense of belonging seem to prevent individuals from identifying more with just one part of the team based on shared salient attributes, possibly resulting in in-group vs. out-group thinking (Tajfel and Turner, 1979). Thus, the current findings support the proposition that team leaders play an important role in managing diversity in work teams and that “the ability of some diverse teams to realize their potential could be tempered because of the individuals who lead them” (Shemla and Wegge, 2019, p. 770). Leaders are in an influential position to model and build a shared identity in diverse virtual teams by highlighting the commonalities and the shared destiny of team members while considering their unique backgrounds (Gibson and Ross-Grubb, 2005; Rosen et al., 2007; Cordery and Soo, 2008). Feeling part of an overarching collective through the actions and behaviors exhibited by an identity leader seems to prevent individual members of a subjectively diverse team from forming categories based on common characteristics among some members (see also Gaertner et al., 2000). However, these findings must be replicated to draw more robust conclusions, as the investigation of identity leadership in virtual teams is still in its infancy. Based on these results, primary conclusions can be drawn that identity leadership appears to be effective in preventing the perception of subgroups in teams where the team members perceive differences to be highly present, promoting satisfaction with the work team, and leading to higher perceived performance. It thus extends previous studies showing that leaders who foster a shared feeling of what “we” stand for by acting as a role model for the team, advancing the group’s interests, and building and providing opportunities to live out the shared identity, are not only critical for positive outcomes in traditional work settings (e.g., Fladerer et al., 2021). They also appear to be important in teams in which team members collaborate primarily through ICT and rarely meet face-to-face due to geographic dispersion. Thus, the present research is in line with recent evidence from the context of the COVID-19 pandemic that also suggests the importance of identity leadership in more remote work settings (Krug et al., 2021).

### Implications

The present study has some interesting implications for virtual team leaders. The findings hint to the point that subjective perception of diversity, meaning that the members are aware of differences among them, as well as the perception of subgroups has a deleterious impact on virtual team outcomes. In the case of performance evaluations, the perception of subgroups being highly prevalent tended to be associated with more negative performance evaluations in subjectively highly diverse teams. Virtual team leaders are thus well advised to counteract the formation of subgroups which might entail an in-group vs. out-group thinking, giving rise to prejudice and stereotypes. This is important as subgroups can undermine the potential of the multifaceted pool of knowledge and resources of diverse virtual teams (e.g., Henttonen and Blomqvist, 2005; Gressgård, 2011).

Prior research has essentially highlighted the power of faultlines in leading to subgroup emergence, especially if geographic locations of some members are aligned with additional surface-level characteristics, like native language or nationality (e.g., Gibbs et al., 2017). The present findings show that not only diversity faultlines can lead to subgroup formation (e.g., Boyraz, 2019), but also the awareness of differences among virtual team members. Leaders should thus be cognizant of their team members’ perceptions of differences and commonalities with their virtual co-workers, paying close attention to first warning signals of beginning subgroup splits. Many recommendations exist as to which strategies employ in order to hinder the emergence of subgroups and to get the best out of their diverse distributed team (Li and Hambrick, 2005; Polzer et al., 2006). Among those recommendations, a considerate selection of potential team members (Polzer et al., 2006), establishing an effective infrastructure to enable communications between all team members (Lau and Murnighan, 2005; Li and Hambrick, 2005) or promoting identification within the whole group (e.g., Polzer et al., 2006; Boyraz, 2019) has frequently been mentioned. The present study extends previous research in that it shows that virtual team members’ perception of team leaders engaging in social identity management behaviors can help mitigate subgroup perception. In this context, the virtual team leader can and should take over the role of a shaper of a shared collective identity to ensure effective team functioning in diverse virtual teams (e.g., Hertel et al., 2005; van der Kamp et al., 2015).

The findings of the present study therefore emphasize the role of an identity leader and at the same time set caution to team leaders which are not capable of developing and advancing a shared team identity. Having a team leader who promotes shared values, represents a model member of a team, creates structures, and provides activities for easier interactions can be a helpful resource, especially if virtually collaborating teams are evaluated by their members as being highly diverse. This also stresses the importance of an accurate training and development of leaders. The so-called 5R Leadership Development Program has been proposed to help leaders understand how to implement strategies to effectively manage social identity (Haslam et al., 2017). Initial evaluations of the 5R program (e.g., Haslam et al., 2017) have provided evidence for its positive impact on the development of identity leadership and thus it might also be interesting to apply to the virtual team setting. In the 5R Program, it is envisioned that once the leader has completed each workshop in which he or she has learned about identity management, the leader will implement this acquired knowledge with his or her team. To ensure that all members are given equal voice, special care and sensitivity is required when some co-located team members participate in these activities in person while other subgroups participate remotely.

### Limitations

First, the cross-sectional design of the study is one obvious limitation of the present research. It is limited by the fact that participants answered the questionnaire at one time point, thus, giving no indication of the development of team processes over time, neither how subgroups are formed or change, nor how perceived diversity changes from the formation of a virtual team until the team has already gained extensive experiences with working together. Additionally, the nonexperimental method used here does not allow to make causal inferences (Levin, 2006), so that the direction of the relationships in this study, for example between perceived diversity and performance or satisfaction ratings, could also be reverse. But still, cross-sectional studies can be an important outset in examining relationships among variables, giving rise to future investigation of the relationships, and can therefore contribute to a primary understanding of the constructs of interest (Spector, 1994).

Second, all data were collected using the same method (an online survey using self-reports), which engenders the concern of common method variance bias (Podsakoff et al., 2003). This means that the variations in responses might have been caused by the same method used for data collection and might thus not reflect true relationships. However, the results of the Harman’s single-factor test (Podsakoff and Organ, 1986) show that, on average, 27% of the variance was explained by the first factor, suggesting that common method variance was not a major problem.

Furthermore, subjective measures are prone to various errors and external influences like mood or previous experiences and are therefore less correct than objective measures (Peters and Karren, 2009). Instead of the subjective team performance measure used here, a more objective measure of team performance (e.g., revenue, sales volume, etc.) might have better accurately reflecting actual performance. As the sample in the present study was heterogeneous regarding their industry sector, asking all participants for the same objective indicator would have been problematic if not impossible. This broad range of contexts and backgrounds of virtual team members precludes the objective assessment of performance on the one side, but, on the other, can be considered a strength of the present research as it increases generalizability of the present findings.

Moreover, it was not possible to calculate diversity on a team level due to a rather small number of team-level responses. Therefore, subjective diversity was examined only on the individual level in this study. Since group composition in terms of team diversity affects not only the individual member, but also team-level processes and outcomes (e.g., subgroup formation; O’Leary and Mortensen, 2010), future studies should aim to examine diversity from a group-perspective. Due to a lack of team data, it was also not possible to calculate objective diversity and compare the effects of specific diversity dimensions on subgroup formation and team outcomes. However, and as already described in the section *Perceived Diversity and Diversity Effects in Virtual Teams*, we decided to use the Perceived Diversity scale to account for general differences virtual team members might perceive, without limiting the investigation to certain dimensions of diversity which team members might or might not be aware of (see also Shemla and Meyer, 2012; Hentschel et al., 2013). Finally, we would like to point out the preliminary nature of the study due to a rather small sample size and possible selection bias, as only a few virtual team members from all contacted companies participated in the survey.

### Future Research

Throughout the discussion, various suggestions for future research have already been presented. In addition, we would like to point out the need for further research regarding the role of identity leadership in the virtual environment and what exact mechanisms or leadership behaviors lead to a feeling of shared identity within virtual teams. In her study on identification-promoting strategies, Sivunen (2006) identified tactics that virtual leaders mentioned to promote a shared identity in their virtual teams. However, it remains unclear whether these tactics are also perceived by team members as promoting identity and in what way these tactics are associated with virtual team performance. Previous research has shown that, for example, we-referencing language (Fladerer et al., 2021) or the leader’s team confidence (Fransen et al., 2016) are associated with performance outcomes. This investigation of leadership behavior or action should be extended to the technology-mediated environment to gain a better understanding of how a shared identity can be established despite more challenging conditions for engaging in shared activities or the reduced presence of social cues.

Establishing a shared sense of “we-ness” is especially difficult in teams composed of diverse and geographically dispersed team members due to the difficulty of making salient the virtual group membership to all team members (Shapiro et al., 2002; Au and Marks, 2012). A shared identity has been claimed to be an important cognitive relationship aspect in virtual teams (Zimmermann, 2011). Research should therefore focus on possible techniques and structures which might help a leader to spread a shared identity (e.g., useful tools or communication media), and when and under what circumstances leaders in virtual team settings are perceived to be identity leaders (e.g., through the use of special team building activities or retreats). Qualitative research designs like interviews with virtual team members could help to get a better insight into certain virtual leadership behaviors which foster a shared social identity and how this, in turn, is associated with virtual team members’ perceived subgroup existence and team outcomes. It would also be worthwhile to investigate how certain dimensions of identity leadership relate to perceived subgroup formation and virtual team outcomes. Future research needs to explore the role virtual leaders (formal or informal ones) can play in building and developing a shared identity among the virtual team members, especially considering the role that ICT play in this process. Therefore, experimental studies could help to investigate which used collaboration or communication tools are most supportive for virtual leaders to help creating, representing and advancing a common identity.

Recent research has emphasized the importance of longitudinal research in studying performance in virtual teams (Contreras et al., 2020). Earlier studies for example have found the tenure of the teammates working together to be essential for team outcomes, and that difficulties in the beginning lose the negative connotation over time (Robert et al., 2009; Gibbs et al., 2017). The herein investigated constructs are dynamic, and future researchers are thus encouraged to extend the current study and follow a more long-lasting view of subgroup formation and identity leadership within virtual teams by implementing a longitudinal research design to examine how leaders’ ability to create a shared identity and subgroup perception evolve over time. This might help to get a step closer to the aim of understanding the mechanisms that underlie the emergence and prevention of subgroups.

Additionally, it would be interesting to investigate the role of informal (identity) leaders, as virtual teams often operate as self-managed teams without a designated leader (e.g., Carte et al., 2006; Ziek and Smulowitz, 2014). Previous research has already tried to identify antecedents and behaviors that lead to the emergence of an individual as a leader (e.g., Yoo and Alavi, 2004; Serban et al., 2015), often focusing on personal traits like extraversion or conscientiousness (e.g., Serban et al., 2015) or communication ability (e.g., Charlier et al., 2016). Charlier et al. (2016), for example, have highlighted the role of communication apprehension and communication ability in emerging as a team leader in dispersed teams. To extent leadership emergence literature, it would be interesting to investigate how emergent leaders gain social support, by examining their ability to represent, cultivate and foster a shared identity within the self-managed virtual team. An interesting avenue for future research would therefore be to examine in an experimental study, whether individuals who represent the particular qualities of the team, advance the teams’ interest, proactively shape and facilitate a shared identity are more likely to emerge as virtual team leaders.

Unfortunately, and as noted earlier, the individual-level data in the current study did not permit to investigate how different dimensions of diversity influence perceived diversity or the awareness of differences. An interesting avenue for further research is thus to investigate how perceived differences or perceived similarities are related to actual measures of diversity or, respectively, similarity in the virtual context, and whether the usage of diverse communication tools influences which characteristics are more closely related to those perceptions. Studies investigating perceived similarity are already common in experimental or offline settings (e.g., Grigoryan, 2020; see Montoya et al., 2008, for a meta-analysis), but are lacking in the virtual context. Especially the reductive capabilities of some ICT might have a special role in forming these diversity perceptions (e.g., Carte and Chidambaram, 2004).

Finally, while the present research provides initial insights about the role of perceived diversity, subgroup formation and identity leadership in virtual teams, more research is needed to replicate the findings at a team level and with larger sample sizes. This would also allow to investigate higher order interactions with possibly interacting factors such as team size and task interdependence. This was not possible with the present sample due to power considerations and so these indicators were only added to our model as control variables (in line with Boyraz, 2019).

## Conclusion

The present research aimed to broaden our understanding of subgroup formation in subjectively diverse virtual teams and to investigate how identity leadership relates to subgroup formation and how this, consequently, is related to team satisfaction and perceived performance. As perceived differences and subgroup formation are often associated with more challenging conditions for effective virtual teamwork, we examined factors that may improve virtual team satisfaction and performance. The present findings demonstrate that individuals who strongly perceive differences among their virtual team colleagues are more likely to also perceive the formation of subgroups more strongly, but only when they do not rate their virtual leaders high on identity leadership. The subjective evaluation of differences is thus a powerful predictor for team outcomes and might be of additional interest when it comes to explaining the member’s satisfaction with the team as well as perceived performance. Moreover, subjective diversity was associated with lower levels of perceived performance only if subgroups were strongly perceived to be present as compared to when they were barely perceived. The present research has thus important implications for virtual team leaders, since fostering a shared sense of “we-ness” might help to overcome the detrimental impact of arising subgroups if the differences within a team are strongly perceived by its members. So far, we know little about social identity management and the actions leaders can take to foster a shared identity in the virtual environment. Future examinations of the underlying mechanisms in the virtual environment can thus help to gain further insights into what leaders can undertake to overcome disruptive effects of perceived team diversity.

## Data Availability Statement

Requests to access the dataset should be directed to corresponding author (KO).

## Ethics Statement

Ethical review and approval was not required for the study on human participants in accordance with the local legislation and institutional requirements. The patients provided their informed consent to participate in this study.

## Author Contributions

HR and KO developed the study concept and designed the research. HR conducted the survey and performed the statistical analyses and drafted the manuscript. HR, HK, and KO edited the manuscript. All authors contributed to the article and approved the submitted version.

## Funding

Philipps University of Marburg provided financial support for the recruitment of participants.

## Conflict of Interest

The authors declare that the research was conducted in the absence of any commercial or financial relationships that could be construed as a potential conflict of interest.

## Publisher’s Note

All claims expressed in this article are solely those of the authors and do not necessarily represent those of their affiliated organizations, or those of the publisher, the editors and the reviewers. Any product that may be evaluated in this article, or claim that may be made by its manufacturer, is not guaranteed or endorsed by the publisher.
